# APTES-Modified Remote Self-Assembled DNA-Based Electrochemical Biosensor for Human Papillomavirus DNA Detection

**DOI:** 10.3390/bios12070449

**Published:** 2022-06-24

**Authors:** Yuxing Yang, Yang Qing, Xudong Hao, Chenxin Fang, Ping Ouyang, Haiyu Li, Zhencui Wang, Yazhen Liao, Haobin Fang, Jie Du

**Affiliations:** College of Materials Science and Engineering, Hainan University, Haikou 570228, China; 20085600210065@hainanu.edu.cn (Y.Y.); 20080500210023@hainanu.edu.cn (Y.Q.); 20197203310048@hainanu.edu.cn (X.H.); 19085204210012@hainanu.edu.cn (C.F.); otping@hainanu.edu.cn (P.O.); 20080500110012@hainanu.edu.cn (H.L.); 20080500110014@hainanu.edu.cn (Z.W.); 21220856000036@hainanu.edu.cn (Y.L.); 21220856000011@hainanu.edu.cn (H.F.)

**Keywords:** cervical cancer, electrochemical detection, 3-aminopropyltriethoxysilane, super sandwich structure

## Abstract

High-risk human papillomavirus (HPV) infection is an important cause of cervical cancer formation; therefore, being able to detect high-risk HPV (e.g., HPV-16) is important for the early treatment and prevention of cervical cancer. In this study, a combination of a 3-aminopropyltriethoxysilane (APTES) modified gold electrode and a super sandwich structure was creatively developed, resulting in the development of a biosensor that is both sensitive and stable for the detection of HPV-16. The electrochemical biosensor possesses a lower detection limit compared with previous studies with an LOD of 5.475 × 10^−16^ mol/L and it possesses a wide linear range from 1.0 × 10^−13^ mol/L to 1.0 × 10^−6^ mol/L (R^2^ = 0.9923) for the target DNA. The experimental data show that the sensor has good stability, and there is no significant decrease in the current response value after 7 days in the low-temperature environment. In addition, the sensor proved to be a powerful clinical tool for disease diagnosis because it showed good interference resistance in complex human serum samples.

## 1. Introduction

Cervical cancer is ranked fourth in the number of cancer cases in women worldwide. According to statistics from the World Health Organization (WHO), 604,000 new cases and 342,000 deaths from cervical cancer were expected to occur in 2020. Approximately 90% of these new cases occurred in low- and middle-income countries [1]. In 2009, the proposal to vaccinate girls against the onset of cervical cancer with HPV was put forward by the WHO. However, this proposal is certainly beyond the reach of existing economies and infrastructure for low- and middle-income countries [2]. The control of cervical cancer is a very important example used as a study of global competition on health issues. It also illustrates the significant differences in public health aspects and healthcare coverage between low- and middle-income countries and high-income countries [3]. For most countries, both HPV-16 and HPV-18 infections are among the most important causes of cervical cancer [4,5]; therefore, it is very important to compete for the detection of high-risk HPV types [6]. However, the key to solving these problems lies in the rapid detection means, as well as the cost of the equipment and the accuracy and convenience of clinical use. This study was carried out with HPV-16 as an example.

In previous studies, several ultra-sensitive methods were developed for the detection of DNA sequences, including quantitative real-time polymerase chain reaction (qRT-PCR) [7], rolling circle replication (RCR) [8], loop-mediated isothermal amplification (LAMP) [9], and sequencing systems [10]. However, these methods have limitations, such as being time-consuming, costly, and expensive, poor stability, and the need for large instruments and equipment, which makes their practical application greatly limited [11]. Therefore, researchers have been working on the development of an electrochemical biosensor with a fast response, high sensitivity, good stability, and high selectivity to achieve the rapid and efficient detection of target detectors. Electrochemical biosensors have been rapidly developed in the field of sensors in the past few years [12].

In 2010, a super sandwich structure electrochemical biosensor was developed by the research of Fan Xia et al. [13]. The structure relies on an amplification strategy for ultra-sensitive detection of the target detectors, and the method is simple, performed at room temperature, and does not require the involvement of enzymes [14]. This proposed structure allows the sensor to be used in electrochemical detection with greatly enhanced current response values. Compared with conventional biosensors, the super sandwich structure allows more DNA to be attached to the electrode, thus increasing the sensitivity. Among other things, experimental results also show that the probe of the super sandwich structure is easier to connect to the electrode and the target DNA than the conventional DNA structure [15,16]. In recent years, the technique has been successfully applied to the highly sensitive detection of pathogens [17], proteins [18,19], nucleic acids [20], and metal ions [21].

3-Aminopropyltriethoxysilane (APTES) is used as a common chemical reagent for surface functionalization [22]. The modification of substances by APTES allows connection between organic, inorganic, and biological molecules [23]. Modification of APTES leads to the formation of APTES membranes, which have an amino group as the terminal group. The amino group is able to react with a variety of groups; therefore, researchers are keen to select APTES as a coupling agent to other chemical bonds in their studies [24]. In previous studies on the immobilization of DNA molecules using APTES SAM, it was found that the good connection performance between APTES SAM and DNA is mainly caused by two reasons. One is because APTES SAM is weakly positively charged, while DNA molecules are strongly negatively charged in solution, and under the action of electrostatic force, DNA molecules are adsorbed onto the electrode surface. The second is because the spatial site resistance formed by the methyl groups on the surface of APTES SAM also helps to immobilize the DNA molecules and stretch them [25,26]. In the fields of medicine [27] and biology [28], APTES is likewise frequently used as a coupling agent for DNA, proteins, and other biomolecules.

Here, we constructed a novel label-free super sandwich structure biosensor based on an APTES-modified gold electrode for ultra-sensitive detection of HPV-16 DNA sequences. The APTES modification allows the sensor to have good stability under complex environments, and its chemical bond modification also improves the efficiency and accuracy of DNA hybridization [29,30]. The super sandwich structure was used as a means of signal amplification that can efficiently improve the sensitive detection of this electrochemical biosensor for the target detectors. The target DNA (TD) used in the experiments was derived from the HPV-16 long terminal repeat sequence [31]. [Ru(phen)_3_]^2+^ is an electrochemical indicator that can be embedded into the double helix structure of DNA and is currently and has been used as a common electrochemical detection reagent [32]. The sensor took a univariate approach to optimize different experimental conditions and also detected the presence of TD in complex human serum samples. The results show that this study can detect HPV DNA with high sensitivity and also provide a new test for the clinical diagnosis of cervical cancer.

## 2. Materials and Methods

### 2.1. Instruments

A CS350H electrochemical workstation (Wuhan Corrtest Instruments Co., Ltd., Wuhan, China) was used for the electrochemical measurements. The electrochemical measurements in this experiment were all performed in a three-electrode system, with the working electrode being a different modified gold electrode, the Ag/AgCl electrode being the reference electrode, and the platinum wire electrode as the auxiliary electrode. All electrodes were procured from Wuhan Gaoshi Ruilian Technology Co., Ltd (Wuhan, China). Scanning electron microscopy (SEM) was performed using TESCAN MIRA LMS (Brno, The Czech Republic) to observe the morphology of the surface after electrode modification.

### 2.2. Reagents

Magnesium chloride (MgCl_2_), sodium chloride (NaCl), potassium chloride (KCl), sodium chloride (NaCl), potassium ferricyanide (K_3_Fe(CN)_6_), and potassium ferricyanide (K_4_Fe(CN)_6_) were bought from Aladdin Co., LTD. (Shanghai, China). The sulfuric acid (H_2_SO_4_) was bought from Xilong Scientific Co., Ltd. (Guangzhou, China). Tris-(2-carboxyethyl)-phosphine hydrochloride (TCEP) was bought from Shanghai Macklin Biochemical Co., Ltd. (Shanghai, China). Tris-HCl buffer (pH 7.4) was purchased from Sanggong Biotech Co. (Shanghai, China). [Ru(phen)_3_]Cl_2_ was purchased from Sigma-Aldrich Trading Co. Ltd. (Shanghai, China). 3-Aminopropyltriethoxysilane (APTES) was bought from Shanghai Maclean Biochemical Technology Co., LTD. (Shanghai, China). Adenosine-5’-triphosphate (ATP) and TE buffer (pH 7.4) were purchased from Shanghai Yuanye Bio-Technology Co., Ltd. (Shanghai, China). Potassium hydroxide (KOH) was purchased from West Asia Chemical Technology Co., Ltd. (Shandong, China). All other chemicals are analytically pure. The ultrapure water (18.25 Ω) used for the experiments was made with Plus-E3 model water purifier of Nanjing Yipu Yida Technology Development Co., Ltd. (Nanjing, China).

The DNA used in this experiment (as shown in Table 1) was synthesized by Sanggong Biotech Co., Ltd. (Shanghai, China), and purified by HPLC. The synthesized DNA was all centrifuged at 4500 rpm/s for 1 min before use, then TE buffer (10 mmol/L Tris-HCl, 1 mmol/L EDTA, pH 7.4) was added and shaken on a vortex shaker for 2 min to fully dissolve, and then stored in the refrigerator at 4 °C for backup.

### 2.3. Electrode Pretreatment

The gold electrode with a diameter of 3 mm was polished with 0.3 μm and 0.05 μm alumina powder on a polishing cloth until the surface was smooth and free of marks, respectively. Then, the electrodes were put into ultrapure water, ethanol, and ultrapure water to allow for ultrasonic cleaning for 5 min, to wash off the excess alumina powder. The electrodes were scanned in 50 mmol/L H_2_SO_4_ solutions using cyclic voltammetry (CV) until stable at voltages of −0.3 V to +1.6 V at 0.1 V/s. The electrodes were rinsed with ultrapure water and dried naturally for subsequent experiments.

### 2.4. Fabrication of Au/APTES

The pretreated electrode was immersed in 1% KOH solution, removed after 10 min, and dried after washing the electrode with ultrapure water. Then, the electrode was immersed in 3% APTES solution and incubated for 1 h, removed and dried for 10 min, and then dried after rinsing the unmembrane-formed APTES with ultrapure water.

### 2.5. Preparation of the DNA Biosensor

Prepare DNA fixation buffer (pH 7.4): TE buffer contains 500 mmol/L NaCl. Prepare DNA hybridization buffer (pH 7.4): TE buffer contains 500 mmol/L NaCl and 1 mmol/L MgCl_2_.

Subsequently, 6 μL of capture probe DNA (CP) (1 μmol/L fixation buffer) was dropped onto the modified electrode and incubated in a humid environment for 12 h. The electrodes were submerged by 10 μL of ATP (10 mmol/L) solution for 1 h. Then, 6 μL samples of different concentrations of target DNA (TD) (in hybridization buffer, pH 7.4) were taken and incubated on the electrode for 2 h. Then, 6 μL of auxiliary DNA (AP1) (1 μmol/L in hybridization buffer, pH 7.4) was taken and incubated on the electrode for 1 h. Finally, 10 μL of freshly prepared auxiliary DNA (1 μmol/L each of AP1 and AP2 in hybridization buffer, pH 7.4) was taken and incubated on the electrode for 2 h. Each incubation step was rinsed with ultrapure water and dried between each incubation step.

### 2.6. Electrochemical Measurement

Cyclic voltammetry (CV) was performed in a 0.1 mol/L KCl solution containing 5 mmol/L [Fe(CN)_6_]^3−/4−^. The voltage was −0.2 V to 0.6 V, and the scan rate was 50 mV/s. Differential pulse voltammetry (DPV) measurements were performed in 10 mmol/L Tris-HCl containing 5 mmol/L [Ru(phen)_3_]^2+^. The scanning potential of DPV was from 0.1 V to −0.6 V with a potential increment of 1 mV, an amplitude of 50 mV, a pulse width of 0.05 s, and a pulse period of 0.1 s. Calculation formula: Signal difference (ΔI) = I_T_ − I_0_, where I_T_ was the peak current in the presence of target DNA (TD) and I_0_ was the peak current in the absence of TD.

## 3. Results and Discussion

### 3.1. Mechanism of the Proposed DNA Biosensor

**Figure 1:**
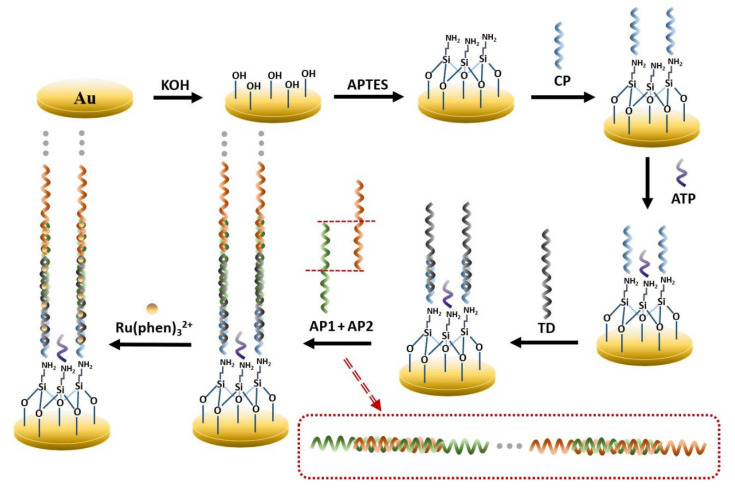
Schematic diagram of APTES-modified super sandwich DNA-based electrochemical biosensor.

The design idea of this electrochemical biosensor is shown in Figure 1. Surface hydroxyl activation was first performed on the bare gold electrode, and then the electrode was modified using APTES, which was chemically bonded to the electrode surface to form an APTES membrane that was used to connect other functional molecules. Since the formed APTES SAM is positively charged and the CP is negatively charged, they will attract each other, and the amino group at the end of the APTES will be attached to the phosphate group on the 5’ of the DNA single-strand, and the CP will be immobilized on the modified electrode. However, the remaining amino sites on the APTES SAM that are not ligated to the CP could still bind to phosphate groups on other single-stranded DNA 5’, thus affecting the selectivity of the experiment. Therefore, the excess sites were closed with ATP in this experiment to ensure selectivity. One end of the TD is then anchored to the electrode by complementary pairing with CP bases and the other end is complementarily paired with AP1, which triggers complementary pairing with AP2 to form a long DNA nanostructure. Many [Ru(phen)_3_]^2+^ are embedded in DNA nanostructures through electrostatic interactions and significantly enhance the electrochemical signal [16,31].

### 3.2. Electrochemical Characterization

**Figure 2:**
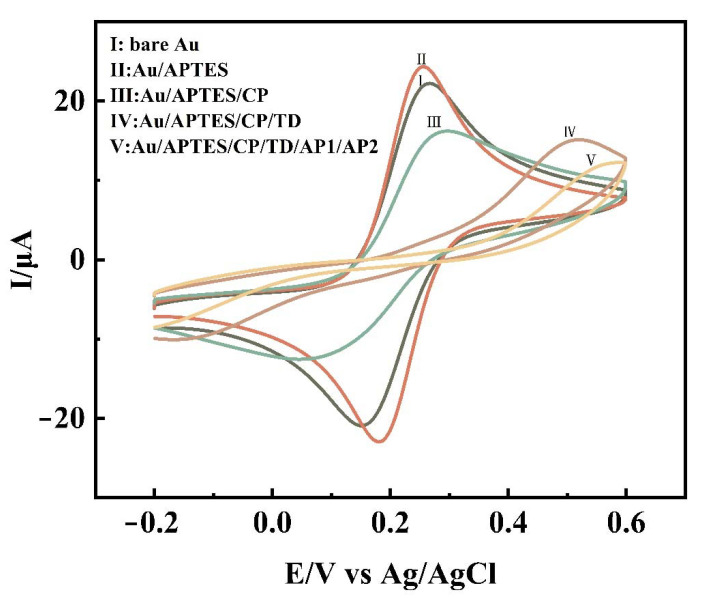
CV at bare Au (**I**), Au/APTES (**II**), Au/APTES/CP (**III**), Au/APTES/CP/TD (**IV**), and Au/APTES/CP/TD/AP1/AP2 (**V**) in 0.1 mol/L KCl solution containing 5 mmol/L [Fe(CN)_6_]^3−/4−^.

CV was used to measure the electrochemical profile after each experimental step to facilitate the observation of whether each step was performed smoothly. Figure 2 shows the cyclic voltammetric curves of the bare gold electrode, Au/APTES, Au/APTES/CP, Au/APTES/CP/TD, and Au/APTES/CP/TD/AP1/AP2. The gold/APTES electrode (Figure 2II) had a significant increase in the redox peak compared with the bare gold electrode (Figure 2I). It was demonstrated that APTES had been successfully modified to the electrode and led to an improvement in electron transfer efficiency. The redox peak of Au/APTES/CP (Figure 2III) was reduced, indicating that the pathway of electron transfer was blocked after the probe was immobilized on the electrode due to the negative charge of the probe. Due to the formation of double-stranded DNA and the generation of repulsion reactions, the redox peak decreased significantly after TD hybridization with CP (Figure 2IV). The introduction of AP1 and AP2 (Figure 2V) triggered the formation of long DNA nanostructures, leading to a further decrease in the redox peak.

**Figure 3:**
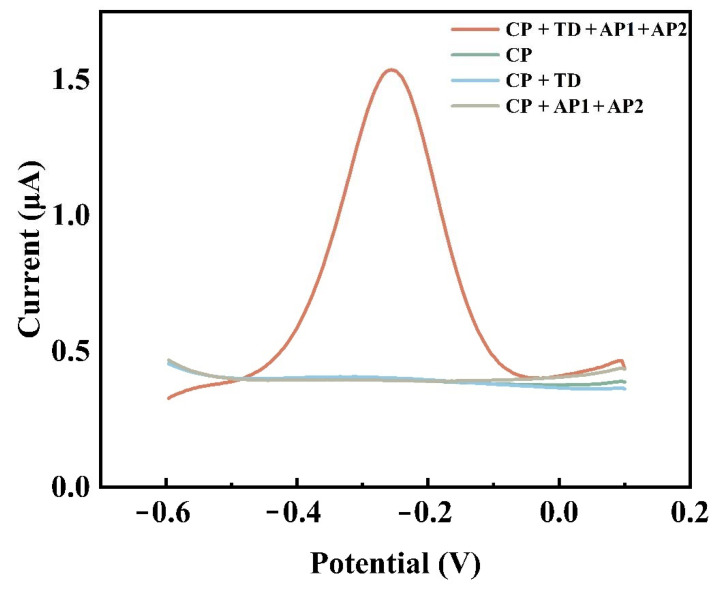
Comparison of DPV response values of the electrodes in the presence of different oligonucleotides. (CP: capture probe, TD: target DNA, AP1: auxiliary probe 1, AP2: auxiliary probe 2.) The concentration of each oligonucleotide was 1 μmol/L.

[Ru(phen)_3_]^2+^ was used as an electrochemical indicator. Figure 3 shows the DPV response of various oligonucleotide-modified gold electrodes. CP was immobilized on the electrode surface through phosphate groups, and there was almost no current response in the absence of TD due to the low binding of [Ru(phen)_3_]^2+^ to CP. After the hybridization of CP with TD, more [Ru(phen)_3_]^2+^ was embedded in the double helix structure of DNA, and a slight increase in the peak can be observed, but it was still negligible. In the absence of TD, but in the presence of AP1 and AP2, the long nanostructures formed by AP1 and AP2 could not be connected to the electrodes and, therefore, the signal response was also barely observed. The long strands of DNA formed by AP1 and AP2 were immobilized on the electrode by hybridization with TD, and the electrochemical reaction was significantly enhanced due to the massive binding of [Ru(phen)_3_]^2+^ to the DNA nanostructures.

### 3.3. Surface Characterization of Modified Electrodes by SEM

**Figure 4:**
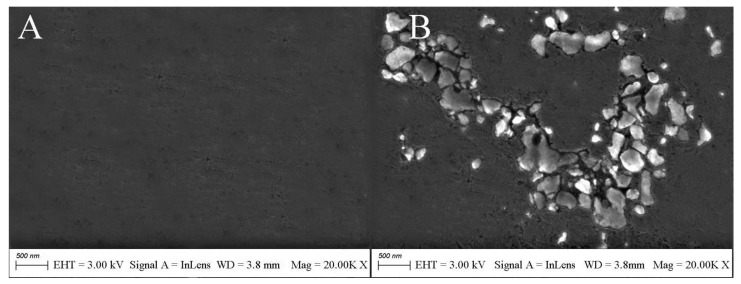
SEM micrographs of (**A**) bare Au and (**B**) Au/APTES electrodes.

The surface morphology of the electrode was analyzed by scanning electron microscopy. The surface of the bare gold electrode was smooth and flat (Figure 4A). The SEM image of the electrode after APTES modification showed island-like morphology (Figure 4B). The reason for the existence of this morphology may be due to the hydrolysis of APTES molecules to produce silanols in the presence of traces of adsorbed water on the electrode surface. If all the methoxy APTES molecules are hydrolyzed, the area occupied by each molecule will contract and a gap will be created between adjacent molecules. APTES molecules in the solution will not be able to insert into the gap due to the presence of spatial site resistance. The existence of the gap also prevents the formation of Si–O–Si covalent bonds between the already hydrolyzed APTES to produce a net-like crosslink. At the same time, due to the short molecular chains of APTES, the van der Waals forces between the molecular chains are weak, and the arrangement of APTES molecules is likely to be skewed and irregular, which eventually leads to the internal structure of the APTES film not being dense and causing the surface to have an island-like shape.

### 3.4. Optimization of the Experimental Conditions

**Figure 5:**
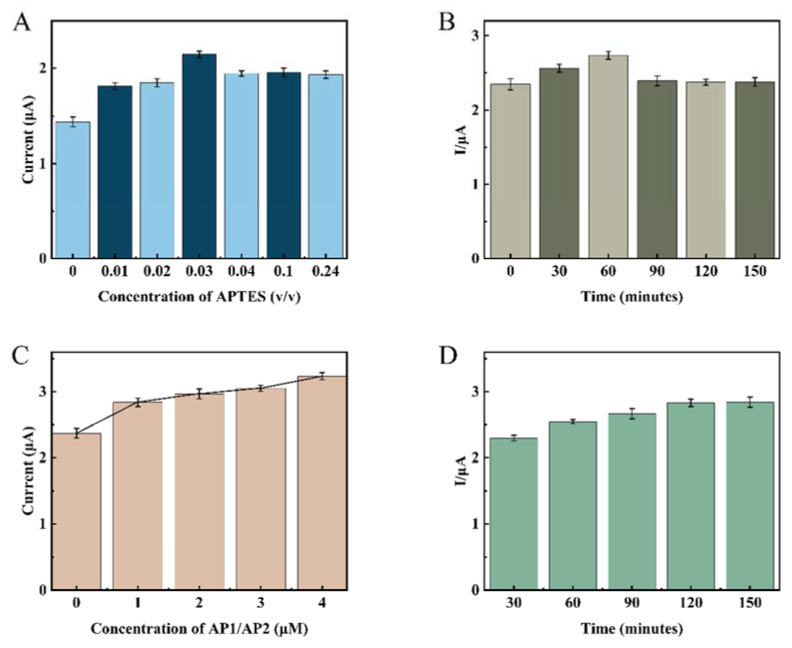
Optimization of each necessary experimental factor. (**A**) Concentration of the APTES; (**B**) APTES reaction time; (**C**) concentration of the AP1 and AP2; (**D**) incubation time of AP1 and AP2. The concentration of CP and TD was 1 μmol/L.

**Figure 6:**
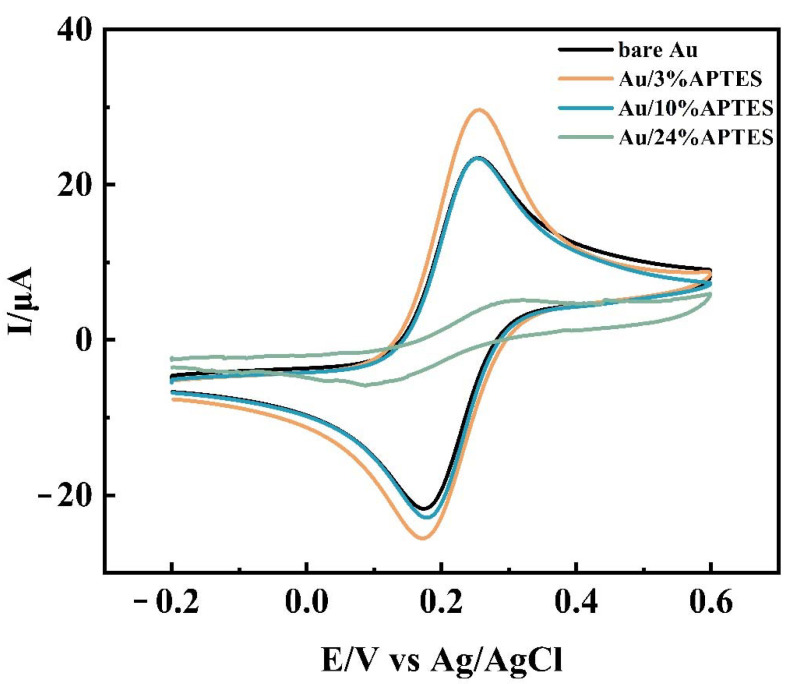
The CV curves of bare gold, Au/3%APTES, Au/10%APTES, and Au/24%APTES electrodes were measured in 0.1 mol/L KCl solution containing 5 mmol/L [Fe(CN)_6_]^3−/4−^.

In order to improve the sensor’s electrochemical performance, various experimental condition parameters need to be optimized, and here, the single variable method was used for condition optimization. Three sets of parallel experiments were adopted for each experiment to facilitate error analysis. First, the modification concentration and modification time of APTES were optimized by DPV and CV measurements. The analysis results showed (Figure 5A) that the increase in APTES concentration led to an increase in the intensity of the current response, which reached a maximum of 3% APTES concentration, and then decreased and leveled off as the concentration continued to increase. Due to the large concentration of APTES, silane will form silanol after hydrolysis in solution, and the continuous condensation of silanol molecules will form a polymer deposited on the electrode surface, which is difficult to remove by conventional washing, affecting the electron transfer efficiency. The CV curves (Figure 6) also verified this statement from another detection means that the electron transfer increased when the electrode was modified using 3% APTES concentration compared with the bare gold electrode. When the concentration increased, the electron transfer efficiency decreased instead, as when the electrode was modified using 10% APTES concentration and 24% APTES concentration. Therefore, in this study, 3% APTES was determined as the experimental modification concentration. Figure 5B shows that the current response of the electrode increases gradually with the increase in the modification time within 1 h. After 1 h, the current response slightly decreased and leveled off, which indicated that there was already an oxide film formation. Therefore, the APTES modification time was determined to be 1 h. As can be seen in Figure 5C, the current response values increased with increasing auxiliary DNA concentration, proving that the one-dimensional nanostructures triggered by AP1 and AP2 can continue to amplify as long as they are present in the system until they are completely depleted. Therefore, from an economic point of view, both AP1 and AP2 were chosen to be 1 μmol/L. After determining the concentrations of AP1 and AP2, their hybridization amplification times were optimized. From Figure 5D, it can be seen that 1 μmol/L of AP1 and AP2 reacted completely at 2 h. Therefore, the incubation time for the auxiliary DNA was determined to be 2 h.

### 3.5. Performance of the DNA Biosensor

**Figure 7:**
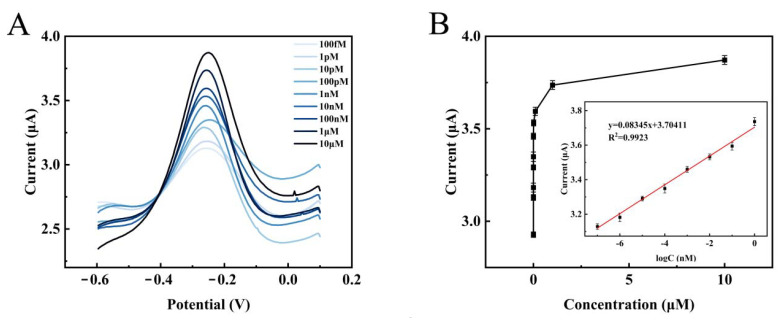
(**A**) DPV curves for different concentration of TD (1.0 × 10^−13^, 1.0 × 10^−12^, 1.0 × 10^−11^, 1.0 × 10^−10^, 1.0 × 10^−9^, 1.0 × 10^−8^, 1.0 × 10^−7^, 1.0 × 10^−6^, and 1.0 × 10^−5^ mol/L). (**B**) Relationship between TD concentrations and DPV intensities. Insert: calibration curve between the logarithm of TD concentrations and ΔI.

Figure 7A shows the DPV performance of the electrochemical biosensor for different concentrations of target DNA under optimal reaction conditions. The current response value of DPV gradually increased as the TD concentration increased from 1.0 × 10^−13^ mol/L to 1.0 × 10^−5^ mol/L. This phenomenon was consistent with the fact that more target DNA can trigger the production of more one-dimensional DNA nanostructures, and the increase in these amplification products allowed more [Ru(phen)_3_]^2+^ to be inserted into the double helix structure of DNA to amplify the signal. Furthermore, as shown in Figure 7B, there was a good linear correlation between the log of TD concentration and ΔI from 1.0 × 10^−13^ mol/L to 1.0 × 10^−6^ mol/L. The regression equation ΔI = 0.08345 log C_TD_ + 3.70411; here was the concentration of TD, and the correlation coefficient of R^2^ was 0.9923. In addition, the calculated limit of detection was LOD = 3σ/S = 5.475 × 10^−16^ mol/L. The detection limit of the sensor in this work was lower than other previously reported biosensors for HPV detection (as shown in Table 2), and the linear range was wide. The above results indicate that the electrochemical biosensor has high sensitivity and wide linear range, suggesting that it shows great potential for clinical applications in detection of the HPV-16 oncogene.

### 3.6. Specificity and Longtime Stability of Biosensor

**Figure 8:**
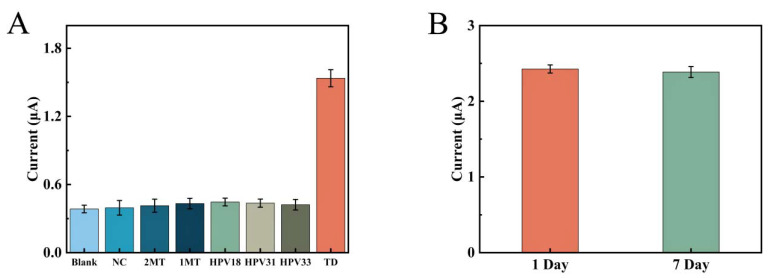
(**A**) Comparison of DPV responses between blank sample sets and different DNA sequences (NC, 2MT, 1MT, HPV18, HPV31, HPV33, TD). (**B**) DPV response before and after the sensor was placed at 4 °C for 7 days. Three sets of parallel experiments were used for error analysis in each experiment.

In this study, seven different DNA sequences were designed to investigate the selective expression of this electrochemical biosensor for unused DNA sequences, including TD, 1MT, 2MT, NC, HPV-18, HPV-31, and HPV-33. Figure 8A shows the selective performance of this sensor. The concentration of TD used here was 1.0 × 10^−6^ mol/L, while the concentrations of all other DNA sequences were 10 times higher. The peak current intensity of the DPV assay containing NC, 2MT, 1MT, HPV-18, HPV-31, and HPV-33 was only slightly higher than that of the blank control. The concentration of TD was 10 times lower than the concentration of other DNA sequences, but the current response value was much higher than the other systems. It indicated that other base sequences cannot be ligated to the CP and also to the one-dimensional nanostructures of AP1 and AP2. This means that this APTES-modified remote self-assembled DNA electrochemical biosensor has excellent selectivity for detecting target DNA and can effectively prevent the influence of other sequences of DNA on the detection results.

In addition, a study of the stability of this electrochemical biosensor was carried out under the above optimal conditions. The electrochemical tests were performed by placing the fabricated sensor at 4 °C for 7 days. As shown in Figure 8B, the measured current value of this sensor did not change much (less than 2%) after 7 days, showing the stability of the electrochemical biosensor.

### 3.7. Analytical Capability of the Resulting Biosensor to Detect HPV-16 in Complex Environments

The ability to detect in complex environments such as serum was also an important indicator. To further test the detection performance of this sensor, recovery studies were performed by adding different concentrations of TD (1, 10, 100 nmol/L) to serum solutions. The experimental results are shown in Table 3. The recoveries ranged from 95.30% to 108.54%, and the RSDs ranged from 3.65% to 6.42%. This indicated that the sensor has good anti-interference performance.

## 4. Conclusions

In this study, a highly sensitive and stable surface-functionalized DNA electrochemical biosensor was successfully constructed for the detection of HPV-16 DNA. The modification of the silicon-based chemical covalent bonding on the bare gold electrode by APTES resulted in a certain degree of improvement in the stability of the sensor, and the current response value of DPV decreased only by less than 2% when left for 7 days. The super sandwich structure is designed to improve the signal detection sensitivity of the sensor by introducing auxiliary DNA for amplification, and the LOD value is as high as 5.475 × 10^−16^ mol/L with a linear range of 1.0 × 10^−13^ mol/L to 1.0 × 10^−6^ mol/L, which is very wide. Remarkably, the sensor is still able to show high immunity to interference in complex serum environments, making it possible for practical applications. In the coming years, the trend in sensors should move toward being more sensitive and portable at low cost. However, this solution provides a powerful and convenient operating platform for DNA detection and an effective approach for early diagnosis of diseases, which is of great value for health issues of global concern. However, the degree of modification of APTES membranes needs to be further optimized and investigated, and for practical applications, a certain degree of improvement and exploration is still needed.

## Figures and Tables

**Table 1:** Sequences of the oligonucleotides used in this study.

Name	Sequence (from 5′ to 3′)
Capture probe (CP)	CCC TCA GAC CCT TAG T
Target DNA (TD)	GTA ATC CAA AAA TTG AAA ACT AAG GGT CTG AGG G
Auxiliary probe 1 (AP1)	TTT CAA TTT TTG GAT TAC CGT GGA CCC CCT CAT
Auxiliary probe 2 (AP2)	GTA ATC CAA AAA TTG AAA ATG AGG GGG TCC ACG
Noncomplementary sequence (NC)	CCT TTT AGT CAG TGT GGA AAT CTC TAG CAG TGG C
Single-base mismatch target (1MT)	GTA ATC CAA TAA TTG AAA ACT AAG GGT CTG AGG G
Two-base mismatch target (2MT)	GTA ATC CAA TTA TTG AAA ACT AAG GGT CTG AGG G
HPV-18	GTA TAT TGC AAG ACA GTA TTG GAA CTT ACA GAG G
HPV-31	CCA AAA GCC CAA GGA AGA TCC ATT TAA A
HPV-33	CAC ATC CAC CCG CAC ATC GTC TGC AAA A

**Table 2:** Comparison between the proposed DPV sensor and other sensors for HPV-16 assay.

Dynamic Line Arrange (mol/L)	LOD (mol/L)	Method	Reference
3.50 × 10^−12^ −3.53 × 10^−11^	1.750 × 10^−12^	DPV	[33]
/	1.750 × 10^−9^	FET	[34]
5.00 × 10^−10^ − 1.00 × 10^−7^	1.500 × 10^−10^	DPV	[35]
1.00 × 10^−14^ − 1.00 × 10^−6^	1.000 × 10^−15^	EIS	[36]
1.00 × 10^−10^ − 2.00 × 10^−7^	3.000 × 10^−11^	ECL	[37]
1.00 × 10^−13^ − 1.00 × 10^−6^	5.475 × 10^−16^	DPV	This work

**Table 3:** Application of the biosensor to detect HPV-16 DNA in serum samples.

TD Added (nmol/L)	Total Found (nmol/L)	Recovery (%)	RSD (%)
1.0	0.953	95.30	3.65
10.0	10.854	108.54	6.42
100.0	103.380	103.38	5.26
